# Supplementary material for: Controlling worm propagation in wireless sensor networks: Through fractal-fractional mathematical perspectives
Source: PLoS One. 2025 Nov 20;20(11):e0335556. doi: 10.1371/journal.pone.0335556 (PMC12633933; doi:10.1371/journal.pone.0335556)
Supplement: Attachment — Submitted filename: Response to Reviewers.pdf [file pone.0335556.s001.pdf]

# Response to the Honorable Editor and Reviewers

Dear Editor,

We appreciate you and the reviewers for your precious time in reviewing our paper and providing valuable comments. It was your valuable and insightful comments that led to possible improvements in the current version. The authors have carefully considered the comments and tried our best to address every one of them. We hope the manuscript, after careful revisions meets your high standards. The authors welcome further constructive comments, if any. Below we provide the point-by-point responses. All modifications in the manuscript have been highlighted in blue.

Sincerely,
The authors

**Detailed Response to Editor**

## Comments:

Controlling Worm Propagation in Wireless Sensor Networks: Through Fractal-Fractional Mathematical Perspectives

This paper proposes a generalized fractional-order model ($SII_1PR$) for analyzing the propagation and containment of worm-based malware in Wireless Sensor Networks (WSNs). The authors build on an earlier SIPR model by introducing an additional "Isolated" compartment ($I_1$) ncorporating fractal-fractional derivatives in the Atangana-Baleanu-Caputo (ABC) sense to capture memory and hereditary effects. They provide theoretical analysis (existence, uniqueness, stability) and a Newton-based numerical scheme to solve the system.

I have the following comments:

1. Lack of real-world validation; no simulations based on actual WSN malware data.
   **Response:** Our model is designed to capture the complex dynamics of malware propagation in wireless sensor networks through careful theoretical validation. The framework is built to reflect well-documented behaviors of real-world attacks. The structure accounts for the non-linear progression of infections due to network constraints, the measurable impact of quarantine protocols, and the critical timing of containment measures—all hallmarks of actual WSN malware outbreaks.

   Through systematic parameter variation and scenario testing, we demonstrate that our model produces behavior patterns consistent with established principles of network infection dynamics. This theoretical validation provides a strong foundation for practical application, offering insights into outbreak progression and defense strategies. The model's flexible design allows for straightforward adaptation when empirical data becomes available, making it a valuable tool for exploring defensive measures and potential attack scenarios in WSN environments.

2. Inadequate explanation of modeling assumptions, particularly for the isolated compartment $I_1$.
   **Response:** The isolated compartment $I_1$ represents nodes that have been completely quarantined through network security measures, with the key assumption that they cannot transmit infection, no ($\beta SI_1$) term. This reflects real-world digital quarantines where compromised nodes are either disconnected or firewalled. The transition rate $\delta_1$ models intrusion detection effectiveness, while $\gamma_2$ represents recovery of isolated nodes. This approach differs from disease models but aligns with network security protocols where isolation can be absolute. We have added to the manuscript ($Section 2.1$) a dedicated paragraph explicitly justifying all compartmental assumptions, including population normalization ($N = 1$) and the $FF - ABC$ operator choice, with references to network security literature supporting these modeling decisions.

3. Overemphasis on formal proofs of standard results, minimal insight or interpretation.
   **Response:** Yes, we have removed overemphasis on the formal proof of results, improved the interpretation all over the paper.

4. Shallow $R_0$ analysis, no stability theorem or global analysis.
   **Response:** We appreciate the reviewer's valuable feedback. Regarding the comment on the "Shallow $R_0$ analysis, no stability theorem or global analysis," we would like to clarify that in Subsection

4.2 ( Theorem 6) of our revised manuscript now, we have indeed provided a detailed global stability analysis based on the basic reproduction number $R_0$. Specifically, we established the global stability of the disease-free equilibrium (DFE) when $R_0 < 1$ and the endemic equilibrium when $R_0$.

5. Numerical method is symbol-heavy without convergence/error analysis or benchmarks.
   **Response:** We thank the reviewer for raising this important point. Regarding the comment on the "Numerical method is symbol-heavy without convergence/error analysis or benchmarks," we acknowledge that in section 6.3, a more detailed discussion of numerical error analysis with some other well-known methods was presented in the manuscript now.

6. Repetitive phrasing and verbose writing add unnecessary length.
   **Response:** The repetitive phrasing was removed from the paper, which helps us to reduce the unnecessary length.

7. Inconsistent or unclear notation ($for example I_\infty vs. I_1$).
   **Response:** Yes, actually it was $I_1$, not $I_\infty$, has been corrected with all other unclear and inconsistent notation now in the revised version.

8. No visual outputs, lacks simulation graphs or figures.
   **Response:** We have now provided some detailed graphs in the numerical simulation section of this paper to strengthen this work.

9. Needs proofreading, grammatical issues and awkward sentences throughout.
   **Response:** Thank you for pointing this out and for your suggestion. We have now rechecked and proofread the whole manuscript for all sought of grammatical issues and improved the English language.

**Recommendations for Improvement:**

1. Add simulation results under various parameter regimes.
   **Response:** Thank you for pointing this out and for your suggestion. The global stability analysis Theorem 6, Subsection 3.2, which we will highlight more prominently. For numerical validation, Figures 11-15 were presented to demonstrate comprehensive simulation results across parameter regimes, and we will supplement these with a formal comparative study and benchmark comparisons against RK4 and Euler methods. These enhancements will further strengthen the study's robustness. We have provided simulation results under different parameters in Figures 11-15.

2. Clarify and justify all modeling assumptions.
   **Response:** Thank you very much for your valuable feedback. All modeling assumptions are clearly justified in Figure 1's flowchart, with dynamical foundations detailed in Section 3.

3. Revise and streamline the numerical method; include convergence or comparative benchmarks.
   **Response:** We have revised and streamlined the numerical method by providing subsection 6.3, on comparative study.

4. Proofread the manuscript carefully for grammar and clarity.
   **Response:** Thank you for pointing this out and for your suggestion. We have now rechecked and proofread the whole manuscript for all sought of grammatical issues to improve the English language and clarity.

5. Include visualizations (foe example $S, I, I_1, P, R$ dynamics for various $R_0$ values).
   **Response:** Yes, we have now included visualizations for $S, I, I_1, P, R$ dynamics for various $R_0$ values in this revised form.

6. Strengthen the $R_0$ analysis with a formal stability result.
   **Response:**Thank you for pointing this out and for your valuable feedback. Regarding the comment on the "Shallow $R_0$ analysis, no stability theorem or global analysis," we would like to clarify that in Subsection 4.2 ( Theorem 6) of our revised manuscript now, we have indeed provided a detailed global stability analysis based on the basic reproduction number $R_0$. Specifically, we established the global stability of the disease-free equilibrium (DFE) when $R_0 < 1$ and the endemic equilibrium when $R_0$ .

Final Recommendation Major Revision. The paper has solid mathematical groundwork but lacks practical validation, clear exposition, and visual support. Needs substantial improvement before it can be considered for publication.
**Response:** Thank you very much for the valuable feedback and suggestion. We have revised and improved our manuscript accordingly.

**Reviewer's Responses to Questions**
Comments to the Author
**1.** Is the manuscript technically sound, and do the data support the conclusions?
The manuscript must describe a technically sound piece of scientific research with data that supports the conclusions. Experiments must have been conducted rigorously, with appropriate controls, replication, and sample sizes. The conclusions must be drawn appropriately based on the data presented.
Reviewer 1 Yes
Reviewer 2 Partly

**2.** Has the statistical analysis been performed appropriately and rigorously?
Reviewer 1 Yes
Reviewer 2: NA

**3.** Have the authors made all data underlying the findings in their manuscript fully available?
The PLOS Data policy requires authors to make all data underlying the findings described in their manuscript fully available without restriction, with rare exception (please refer to the Data Availability Statement in the manuscript PDF file). The data should be provided as part of the manuscript or its supporting information, or deposited to a public repository. For example, in addition to summary statistics, the data points behind means, medians and variance measures should be available. If there are restrictions on publicly sharing data—e.g. participant privacy or use of data from a third party—those must be specified.
Reviewer 1 Yes
Reviewer 2 Yes

**4.** Is the manuscript presented in an intelligible fashion and written in standard English?
PLOS ONE does not copyedit accepted manuscripts, so the language in submitted articles must be clear, correct, and unambiguous. Any typographical or grammatical errors should be corrected at revision, so please note any specific errors here.
Reviewer 1 Yes
Reviewer 2 No

**Review Comments to the Author**
Please use the space provided to explain your answers to the questions above. You may also include additional comments for the author, including concerns about dual publication, research ethics, or publication ethics. (Please upload your review as an attachment if it exceeds 20,000 characters)

# Detailed Response to Reviewer 1

Manuscript $D : PONE - D - 25 - 20163$ Title: Controlling Worm Propagation in Wireless Sensor Networks: Through Fractal-Fractional Mathematical Perspectives

This manuscript presents a mathematical modeling approach to understand and control worm propagation in Wireless Sensor Networks (WSNs) by extending the classical $SIPR$ (Susceptible-Infectious-Protected-Recovered) model. The authors incorporate a novel compartment for isolated nodes ($I_1$) and apply a fractal-fractional operator (in the Atangana-Baleanu-Caputo sense) to better capture memory and hereditary characteristics of the system. The model is analyzed through fixed-point theory, existence and uniqueness proofs, stability analysis, and is numerically solved using a Newton polynomial-based scheme. The work also computes the basic reproduction number $R_0$ and provides sensitivity analysis for key parameters. The proposed model adds valuable complexity to the classical epidemic framework in WSNs by incorporating isolation dynamics and fractal-fractional derivatives. This is a meaningful extension, particularly for systems with memory effects. The model is relevant and timely for enhancing

cybersecurity in sensor networks. While the work demonstrates potentially valuable contributions, the manuscript requires significant revisions before it can be considered for publication.

1. The manuscript is generally well-structured but would benefit from more clarity in certain sections: Consider breaking down complex derivations into subsections or appending them in supplementary material. Not all variables or acronyms (for example FFD, ABC, HUS) are defined upon first use; ensure full clarity for readers less familiar with fractional calculus. The introduction provides a broad overview of WSNs and malware threats but lacks a focused literature review. The novelty of the proposed approach compared to previous models should be articulated more clearly.
**Response:** To improve clarity, we have streamlined the paper in Section by breaking it into smaller subsections and moving detailed steps to the supplementary material in subsections. These changes enhance readability while maintaining rigor. Thank you for your valuable feedback.*we have carefully reviewed the manuscript and ensured that all variables and acronyms (including FFD (Fractal Fractional Derivative), ABC (Atangana-Baleanu-Caputo), and HUS (Hyers-Ulam Stability) are now explicitly defined upon their first use in the text. These clarifications will improve readability and accessibility for readers less familiar with fractional calculus concepts. We have significantly strengthened the manuscript by expanding the Introduction and adding literature-related works to this work to systematically review prior WSN malware detection models, explicitly highlighting gaps and novelty of our work.

2. The current literature review, though rich in references, is more descriptive than analytical. It would be strengthened by thematically grouping prior models (e.g., classical ODE models, fractional models, models with isolated compartments) and explicitly stating what limitations each had that the current study addresses.
**Response:** Thank you for pointing this out and for your insightful suggestion. We have restructured the literature review to thematically organize prior works (e.g., classical ODEs, fractional models, compartmental approaches) and now explicitly highlight their key limitations, particularly gaps that our study addresses. This revision strengthens the analytical depth and justifies our methodological innovations.

3. The addition of the isolated compartment $I_1$ is intuitive, but its real-world implementation should be justified. For example, how practical is it to isolate infected nodes in a WSNs context, and what mechanisms (software, hardware) could facilitate this?
**Response:** The isolated compartment $I_1$ reflects practical WSNs security measures where infected nodes are forcefully disconnected (but retained in the network for potential recovery). Modern systems deploy lightweight intrusion detection to identify threats and automatically isolate compromised nodes. Adaptive protocols reroute data flows dynamically, while hardware-based deactivation (e.g., remote sleep commands) enforces containment. This strategy balances security with operational continuity, demonstrating $I_1$'s real-world viability for worm control.

4. The numerical scheme using Newton interpolation is appropriate, though a brief justification for selecting this method over alternatives (e.g., finite difference or Runge-Kutta methods) would be helpful.
**Response:** We employed Newton polynomials to solve our fractional order WSN model because they are particularly well suited for capturing the non-local and memory-dependent nature of fractional calculus, which is essential for modeling sensor network dynamics. Unlike finite difference methods, which require structured grids and struggle with historical dependencies, or Runge-Kutta schemes that are designed for integer order ODEs. Newton interpolation naturally accommodates irregularly spaced data points common in WSNs while avoiding the singularity issues typical of grid-based approaches. Additionally, this method provides an optimal balance between accuracy and computational efficiency, as demonstrated in our results ($Subsection 6.3$). We have added a brief justification in the revised manuscript to clarify this choice.

5. The simulations are not detailed in this excerpt. If present in the full version, ensure they clearly demonstrate the dynamic differences between classical and proposed models.
**Response:** We thank the reviewer for their insightful comments. All modeling assumptions are rigorously justified in Figure 1 and Section 3 of this work. Theorem 6 (Subsection 3.2) provides a complete global stability analysis. Regarding simulations, Figures 11-15 in the full manuscript explicitly compare dynamic behaviors between classical and proposed models under varied parameter regimes, demonstrating key differences.

6. The manuscript requires moderate English language editing. There are several syntactic and grammatical issues that may hinder comprehension. For example, the phrasing is at times redundant or overly formal. A professional language revision is strongly recommended.
   **Response:** Thank you for pointing this out and for your insightful comments. We rechecked and proofread the whole manuscript for all sought of grammatical issues to improve the English language and clarity.

7. Clearly define all parameters in Table 1 and ensure consistent notation throughout.
   **Response:** Thanks for your suggestions, we have now clearly defined all parameters in Table 1 .

8. Add a paragraph at the end of the introduction to outline the paper's structure.
   **Response:** We sincerely appreciate your valuable suggestion. In response, we have added a paragraph at the end of the introduction section that outlines the paper's organizational structure.

9. Ensure all equations are properly referenced and integrated into the discussion.
   **Response:** All equations are properly referenced and integrated into the discussion in the revised form of the article now.

10. Figures and tables, must be explicitly cited and discussed in the main text.
    **Response:** Yes, all figures and tables are cited and discussed in the main work now.

11. Consider including a summary of key contributions as bullet points for clarity.
    **Response:** We have now included a concise bullet-point summary of our key contributions in the Conclusion section for greater clarity

Although, the manuscript demonstrates significant potential and rigor, it requires substantial revisions to enhance its clarity, contextual grounding, and presentation. I encourage the authors to address the above comments carefully.
**Response:** Thank you very much for the valuable comments and suggestions. We have revised and improved our manuscript accordingly.

## Detailed Response to Reviewer 2

This study has presented a mathematical model for analyzing worm-spreading dynamics in wireless sensor networks. It has extended the traditional Susceptible-Infected-Protected-Recovered model by incorporating an isolated compartment. The ABC fractional derivative has been employed to represent memory-influenced behavior and network-wide interactions. I would recommend publication after the issues below are addressed.

1. Huge editing is required for the English language throughout the manuscript due to numerous grammatical and spelling mistakes.
   **Response:** Thank you very much for your valuable feedback and comment. We have rechecked and proofread the whole manuscript for all sought of grammatical issues and improved the English language.

2. The full title and short title of the manuscript are the same; however, the short title must be shorter than the full one.
   **Response:** We have now revised the short title to be appropriately concise and distinct from the full title, as highlighted in the manuscript (Section 3). The modification has been made throughout the document to ensure consistency.

3. Two authors are marked as corresponding authors on the title page. One of them should be removed. Also, two affiliations are numbered as 3; the second one should be numbered as 4.
   **Response:** Thank you for pointing this out. Therefore, we have removed one of the corresponding authors and corrected the affiliations numbers.

4. The manuscript is prepared using the Elsevier LaTeX template. However, PLOS ONE is not an Elsevier journal, and the phrase at the bottom left of the title page should be deleted.
   **Response:** Thank you for pointing this out. Therefore, we have now used the PLOS ONE template.

5. No citations should be inserted in the abstract. Also, no more than three citations should be inserted in a single statement. The extra ones should be removed or moved to other appropriate sentences. Besides, the in-text citations should be numbered in the order of their appearance.
**Response:** We appreciate your valuable comment. Following the journal's guidelines, we have carefully revised the abstract to ensure it contains no citations. Additionally, we have adjusted the in-text citations to ensure no single statement includes more than one reference. Extra citations have either been removed or relocated to more appropriate sentences. All in-text citations have also been renumbered sequentially in the order of their appearance.

6. All citations of the third and fourth paragraphs of the introduction are inserted at the end of the paragraph. However, they should be distributed in the paragraph and inserted in relevant places. In addition, reference 25 is not cited in the text but is listed in the bibliography. This issue should be solved.
**Response:** We appreciate your valuable comment. As suggested, we have redistributed the citations in the third and fourth paragraphs of the Introduction, placing them at relevant points within the text rather than at the end of the paragraphs. Additionally, we have removed Reference 25 from the bibliography. These revisions ensure proper citation placement and consistency with the journal's guidelines.

7. All abbreviations should be accompanied by their complete forms when first used in the abstract and the main text. Afterward, all complete forms should be replaced with their abbreviations.
**Response:** Yes, we agree with you. Now, all abbreviations in both the abstract and main text are properly defined upon first use, followed by their abbreviated forms in subsequent mentions. This improves clarity for the journal's guidelines.

8. Equation (1), Table 1, and their associated text should be removed from the introduction and inserted in Section 3, with any necessary changes made.
**Response:** Thank you for your valuable suggestion regarding the placement of Equation (1) and Table 1. As recommended, we have moved these elements from the Introduction to Section 3. We believe this reorganization improves the paper's structure by presenting methodological details in the appropriate section.

9. A paragraph should be added to the end of the introduction that describes the manuscript's structure section by section.
**Response:** We sincerely appreciate your valuable suggestion. In response, we have added a paragraph at the end of the introduction section that outlines the paper's organizational structure.

10. Table captions should be inserted above them, not below them.
**Response:** Done as directed.

11. Only the mathematical symbols representing matrices should be written in bold; the rest should not be in bold.
**Response:** The mathematical symbols representing matrices have been written in bold, while the rest are not bold in the revised version.

12. The numbering of all Assumptions, Definitions, Lemmas, Remarks, and Theorems should be double-checked.
**Response:** Thank you for pointing this out and for your suggestion. Therefore, we have thoroughly rechecked and revised the numbering of all Assumptions, Definitions, Lemmas, Remarks, and Theorems.

13. All '$I_\infty$' should be replaced by '$I_1'$. The right-hand side of Equation (13) should be identical to Equation (1). In the last line of Equation (15), a ')' is misplaced. In the proof of Theorem 5, when subtracting the kernels, the fourth line should be omitted, and the first minus sign in the fifth line should be deleted. In the fourth line of Equation (19), the '$||.||'$ should be applied only to the kernel (like other lines), and the '$\{\}'$ should be removed. In all equations, the beta function should be denoted by '$\beta'$, not 'B'. In the equation above Equation (21), the '$l_1'$ on the right-hand side should have the same subscript as the 'F' on the left-hand side. In the equation below, 'Now, putting ...' on page 11 and the third line of Equation (23), '$S_1'$ should be corrected as $'S_2'$. In the fifth equation on page 12, '$Y_3'$ should be corrected as '$Y_4'$ or '$Y_5'$, and the equations for '$K_4'$ and '$K_5'$

should be separated. The first minus in the fourth line of Equation (23) should be removed. On the right-hand side of Equation (24), a '+' is missing after '$\mu$'. There is an extra ',' in the equation below Equation (34).
**Response:** We have addressed all the reviewers' suggestions in the revised version. All instances of $I_\infty$ have been replaced with $I_1$, and Equation (13) has been adjusted to match Equation (1) (now Equation (12) in the revised version, as suggested by Reviewer 2). In Equation (15), a missing parenthesis was added at its particular position. The proof of Theorem 5 (now Theorem 3 after renumbering) was corrected by removing a fourth line and a minus sign from the fifth line. Notation consistency was improved using $\beta$ instead of B for the beta function and fixing '$||.||'$ placement in Equation (19). In the fourth line of Equation (19), the '$||.||'$ has now applied only to the kernel $\mathbf{K}_2\Big(\mathbf{I}(\mathtt{t})\Big)$, and the '$\{\}'$ has been removed. I think that in Equation (21), $\Upsilon_1$ on the right-hand side should have a different subscript, because $\Upsilon_1 and \Upsilon_2$ are two different functions. Corrections were also made in Equations (23) and (24), including fixing $S_1$ to $S_2$, adjusting signs, and adding a missing '+'. On Page 12, the fifth equation '$Y_3'$ have been corrected as '$Y_4'$(In case of $K_4$) and '$Y_5'$ (in case of $K_5$), and also define in separated equations. Yes first minus in the fourth line of Equation (23) has been removed. Yes, on the right-hand side of Equation (24), a '+' was missing after '$\mu'$, and has been placed now, and an extra comma below Equation (34) was removed.

14. The second line of Equation (29) and Equation (30) do not agree with the second line of Equation (1). Which one is correct? If Equation (29) is correct, Equation (1) and all following equations should be revised.
**Response:** Yes, the second line of Equation (29) and Equation (30) do not agree with the second line of Equation (1). There was a typo in Equation (1) (is now Equation (12) in the revised paper); the parameter $\delta_1$ was missing in Equation(1), which was corrected in the revised version. while Equation (29) was correct, and also the remaining equations.

15. There are two equations in Equation (34). Which one is correct? The correct one should be kept, and the other should be omitted. Additionally, it seems that the sigma operator should be applied to all expressions until the end of the equation. Consequently, the parentheses of this equation and the following ones should be reordered.
**Response:** We have removed the duplicate equation in Equation (34) and kept only the correct version. Additionally, we have ensured that the sigma operator $\sum$ applies to all relevant terms until the end of the equation (highlighted in the revised version).

16. The plotted titles should be removed from the figures. Moreover, the x-axis label of all figures should be 'Time (days)'. Furthermore, the font name and size of all figures should be identical.
**Response:** We have removed plotted titles from figures, the x-axis of figures is labeled as "Time (Days)", and the font name and size are now identical in the revised version.

17. In Figure 2a, the y-axis labels should be changed to 'I'. All the subscripts in the legends of panel b are 1; some of them should be changed to 2. The y-axis labels of all panels in Figure 4 should be 'P'.
**Response:** Thank you for your valuable suggestion. The y-axis of Figure 2a (is now Figure 6a in the revised paper and inclusion of other figures by reviewers) is now labeled as I(t), and we corrected subscripts in the legends of panel b accordingly and also corrected the labels for panel 4b too (4b is now figure 8b in the revised paper and inclusion of other figures by reviewers ).

18. Figure 6 is a single panel, so the '(a)' in its caption should be removed and its text should be merged into the figure's caption.
**Response:** The '(a)' in figure 6 (figure 10 in the revised version) has been removed, also its text has been merged in the figure's caption.

19. What do the dashed lines in Figure 6 mean?
**Response:** The dashed lines were used to distinguish the graphs of each compartment from one another. Meanwhile, we have used the uniform solid lines of different colors to distinguish them.

20. 'Future Works' and 'Authors Contributions' sections should be included in the manuscript.
**Response:** Thank you for your suggestion. We have now included 'Future Works' and 'Authors Contributions' sections.

We express our sincere gratitude to the reviewers for their valuable feedback and insightful suggestions. These comments, which addressed our work's concerns in detail and highlighted areas requiring further development, have greatly strengthened the manuscript's rigor and clarity.

Yours sincerely,
The authors
